# Genetic dissection of fatty acid components in the Chinese peanut (*Arachis hypogaea* L.) mini-core collection under multi-environments

**DOI:** 10.1371/journal.pone.0279650

**Published:** 2022-12-30

**Authors:** Xiaojing Zhou, Huaiyong Luo, Bolun Yu, Li Huang, Nian Liu, Weigang Chen, Boshou Liao, Yong Lei, Dongxin Huai, Pengxia Guo, Weitao Li, Jianbing Guo, Huifang Jiang

**Affiliations:** Key Laboratory of Biology and Genetic Improvement of Oil Crops, Ministry of Agriculture, Oil Crops Research Institute of the Chinese Academy of Agricultural Sciences, Wuhan, Hubei, China; ICAR-Indian Institute of Pulses Research, INDIA

## Abstract

Peanut (*Arachis hypogaea* L.) is an important source of edible oil and protein for human nutrition. The quality of peanut seed oil is mainly determined by the composition of fatty acids, especially the contents of oleic acid and linoleic acid. Improving the composition of fatty acids in the seed oil is one of the main objectives for peanut breeding globally. To uncover the genetic basis of fatty acids and broaden the genetic variation in future peanut breeding programs, this study used genome-wide association studies (GWAS) to identify loci associated with target traits and developed diagnostic marker. The contents of eight fatty acid components of the Chinese peanut mini-core collection were measured under four environments. Using the phenotypic information and over one hundred thousand single nucleotide polymorphisms (SNPs), GWAS were conducted to investigate the genetics basis of fatty acids under multi-environments. Overall, 75 SNPs were identified significant trait associations with fatty acid components. Nineteen associations were repeatedly identified in multiple environments, and 13 loci were co-associated with two or three traits. Three stable major associated loci were identified, including two loci for oleic acid and linoleic acid on chromosome A09 [mean phenotypic variation explained (PVE): 38.5%, 10.35%] and one for stearic acid on B06 (mean PVE: 23%). According to functional annotations, 21 putative candidate genes related to fatty acid biosynthesis were found underlying the three associations. The allelic effect of SNP A09-114690064 showed that the base variation was highly correlated with the phenotypic variation of oleic acid and linoleic acid contents, and a cost-effective Kompetitive allele-Specific PCR (KASP) diagnostic marker was developed. Furthermore, the SNP A09-114690064 was found to change the cis-element CAAT (-) in the promoter of *ahFAD2A* to YACT (+), leading dozens of times higher expression level. The enhancer-like activity of *ahFAD2A* promoter was identified that was valuable for enriching the regulation mechanism of *ahFAD2A*. This study improved our understanding on the genetic architecture of fatty acid components in peanut, and the new effective diagnostic marker would be useful for marker-assisted selection of high-oleic peanut breeding.

## Introduction

Peanut (*Arachis hypogea* L.) is an important oilseed as well as economic crop in more than 100 countries of Asia, Africa, America. In 2020, world production of peanut (with shell) was 53.64 m tons harvested from an area of 31.57 m ha (FAO 2020) [1]. Peanuts are usually used for extraction of edible oil, consumed as seeds, processed food products, livestock fodder and green manure. The peanut oil accounts 40–56% of the dry seed weight [2] and the fatty acid composition is important among the final quality of the oil. Improving the fatty acid composition of peanut oil is one of the important goals for cultivar breeding.

The fatty acids in peanut oil are mainly oleic acid (C18:1) and linoleic acid (C18:2), accounting for about 80% of the total [3, 4]. Several benefits of oleic acid drive the breeding effort toward producing high oleic peanuts. Oleic acid has 10-fold higher auto-oxidative stability than linoleic acid [5]; therefore, high oleic acid and low linoleic acid peanut has a longer shelf life than normal peanut [6]. In addition, oleic acid was shown to decrease blood low-density lipoprotein (LDL) levels, suppress tumorigenesis, and ameliorate inflammatory diseases [7–9]. The first high oleic acid peanut F435 which was also the most famous high oleic acid mutant showed a 448G>A mutation in the *ahFAD2A* coding region of the mutant [10, 11]. Many high oleic acid peanut cultivars have been developed through the 448G>A locus of *ahFAD2A* gene. Considering the complexity of the gene regulation, new loci need to be identified to provide more options about improving the oleic acid content in peanut seeds. The other six peanut fatty acid components, palmitic acid (C16:0), stearic acid (C18:0), arachidic acid (C20:0), behenic acid (C22:0), lignoceric acid (C24:0), and gadoleic acid (C20:1), account for 20% of the total fatty acids [3, 4]. The six fatty acids are all saturated fatty acids except for C20:1. High saturated fatty acid intake is usually thought to be a major cause of elevated cholesterol, triglyceride and LDL-C, which increases the risk of cardiovascular disease. However, not all saturated fatty acids are considered to be absolutely unhealthy, studies have shown that stearic acid is a healthy substitute for trans fatty acid in food manufacturing, and high levels of stearic acid may reduce the risk of atrial fibrillation [12, 13].

Fatty acid components are quantitative characters, which are easily influenced by environment. So far, the studies on the quantitative trait loci (QTL) mapping of fatty acids in peanut are limited, and most of the related QTLs have been obtained by linkage mapping using hybrid segregating populations [4, 14, 15]. But the constructed linkage maps usually include only hundreds of markers, with the exception of three linkage maps that include over 1000 markers [15–17]. The low density of the linkage maps are unable to provide precise information on the numbers and locations of QTLs controlling the fatty acid traits. Compared to linkage mapping, genome-wide association studies (GWAS) do not need to take many years to construct mapping populations. It can evaluate multiple alleles at a single locus and provide a higher mapping resolution. With the rapid development and low cost of next-generation sequencing, GWAS based on millions of markers has emerged as a powerful approach for facilitating the genetic dissection of important traits and accelerating marker-assisted breeding [18–20]. In addition, the genome sequence resources of *A*. *duranensis*, *A*. *ipaensis* [21–23] and *A*. *hypogaea* [24–26] were reported, providing important foundations for exploring the molecular basis of phenotypic variations in peanut.

Our recently study reported identification of 105814 SNP markers from genotyping-by-sequencing (GBS) sequencing data of the Chinese peanut mini-core collection [27]. This study focused on dissection of genetic basis of fatty acids in peanut. The genomic loci were systemically identified for eight fatty acid components in multi-environments. Candidate genes underlying the association loci with large phenotypic variance explained (PVE) were sought and the sequence variations were investigated in gene regions contributing to the phenotypic differences. The deep dissection will enhance our understanding of the genetic architecture of the fatty acids and provide useful information for peanut breeding.

## Materials and methods

### Field experimentation

A panel of 250 germplasm resources of the Chinese peanut mini-core collection was used for genome-wide association study (GWAS) in this study. The detail information including their origins and botanical subspecies was published previously as supplementary materials [27]. In 2015 and 2016, the population was grown in the fertile alluvial soil of the experimental farm of Nanchong Academy of Agricultural Sciences, Nanchong, Sichuan, China (30°80’N, 106°06’E), and in the fertile loam of the experimental field at Oil Crops Research Institute of Chinese Academy of Agricultural Sciences, Wuhan, Hubei, China (30°35’N, 114°33’E). The four experimental environments of Nanchong in 2015, Wuhan in 2015, Nanchong in 2016 and Wuhan in 2016 were designated as “E1, E2, E3, E4", respectively. Field trails were performed using a random block design with three replications in each environment. Each plot had one row of 1.5 m length. The distance between each row was 30 cm, and there was 10 cm between plants in each row. After sowing, the field was irrigated to ensure normal seed germination. Continuous monthly meteorological data of growth periods for two climate locations in 2015 and 2016 (including the average lowest ~ highest temperature, accumulated sunshine, average humidity, precipitation) were shown in **S1 Table in S2 File**. Field management followed the recommended cultural practices with manual weeding and no spraying of insecticides and fungicides. After 130 days of sowing, the plants were harvested except marginal ones.

### Fatty acid profiling

After the harvested pods were dried, the contents of fatty acids of the population were measured, including C16:0, C18:0, C18:1, C18:2, C20:0, C20:1, C22:0 and C24:0. We randomly selected 10 mature and plump seeds of each plot and ground into powder. Approximately 20 mg powder in triplicate was used for extraction and measurement of fatty acids. First, 20 mg seed powder was blended with 1 mL petroleum ether. Then 400 μL of 0.5 mol/L sodium methoxide (NaOCH3) in methanol solution was added to convert fatty acids to methyl esters. After 1 hour, the organic layer containing the methyl esters was transferred to an autosampler vial for gas chromatograph analysis. Fatty acid composition was determined using an Agilent 7890B gas chromatograph equipped with a flame ionization detector (FID) and an autosampler (Agilent Technologies, USA). Peak separation was carried out on DB-23 capillary column (30 m×0.25 mm I.D., 0.25 μm film thickness, Agilent Technologies, USA). One μl of sample was injected at a 60:1 split ratio onto the column maintained isothermally at 220°C. The temperature of the inlet and detector were set at 260°C and 280°C, respectively. Fatty acid composition was determined by calculating relative peak areas.

### Statistical analysis

Statistical analyses for phenotypic data of fatty acid composition in four environments were performed using IBM SPSS Statistics software (version 21). The broad-sense heritability for each trait across environments was calculated according to Hallauer and Miranda (1998) [28] as *H*^*2*^ = *σ*^*2*^_*g*_/(*σ*^*2*^_*g*_+ *σ*^*2*^_*ge*_/ *n* + *σ*^*2*^_*e*_/ *nr*), where *σ*^*2*^_*g*_ was the genetic variance, *σ*^*2*^_*ge*_ was the variance due to *G* × *E* interaction, *σ*^*2*^_*e*_ was the residual error, *n* was the number of environments, and *r* was the number of replications within environment. Correlation coefficients between traits across four environments were also performed on IBM SPSS Statistics software (version 21). The major allele frequency, genetic diversity and polymorphism information content (PIC) were calculated using PowerMarker V3.25 software [29].

### GWAS and candidate gene scan on fatty acids

A total of 105814 high-quality SNPs with minor allele frequency (MAF) ≥ 0.05 in the Chinese peanut mini-core collection [27] were used for genome-wide association analysis. To identify association loci for contents of eight fatty acids in multi-environments, GWAS was conducted using genome-wide efficient mixed model association (GEMMA) software [30]. Mixed linear model (MLM) was selected because of its best performance to eliminate false positives. MLM was coupled with previously estimated principal component analysis matrix and kinship matrix as random effects [27]. We used *P* = 0.05/ (total SNPs) (i.e., 0.05/105814, 4.73×10^−7^) as the genome-wide significance threshold after Bonferroni correction [31]. In order to highlight the significant sites in Manhattan plot, a negative logarithm base of 10 was used to convert the *P* value (-log104.73×10–7 = 6.33). The Manhattan and quantile–quantile (Q-Q) plots of GWAS results were generated in R program. The phenotypic variation explained (PVE) of significant loci were estimated by ANOVA as described [32]. To identify the putative candidate genes underlying the stable major associated SNPs, approximately 1.3Mb decay distance of linkage disequilibrium reported by our previous study was used to search genes [27]. The genes were selected as candidate genes in the genomic regions if they encode components of metabolic or signaling pathways known to be related to the fatty acids.

### Validation of variation in the diagnostic marker

The base variation of SNP A09_114690064 was confirmed by PCR amplification and sequencing. The specific primers were designed upstream and downstream of the locus were: *ahFAD2A*-F: CATTGCACAAGGCAACCGAA, *ahFAD2A*-R: CGAACGGCTATGAAACCAGC. The cis-acting regulatory DNA elements was analyzed using http://www.dna.affrc.go.jp/PLACE/. The KASP marker was developed using the flanking 100 bp sequences of the SNP variant [33]. Two allele-specific forward primers and one common reverse primer were designed and synthesized. The KASP primers were as following. Primer_Allele X: GTAAAATAAATAGTTCCAGTTTAACTTAAGC, Primer_Allele Y: GTAAAATAAATAGTTCCAGTTTAACTTAAGT, Primer_Common: CCAAGAGTCTCTAAAAATAGTGCTAGCAT. Sequences of the KASP primers do not include the tail sequences that interact with the fluor-labelled oligos in the KASP reaction.

### qRT-PCR analyses

Three materials (Guangdehuasheng, FDRS10 and Anshanxiaohuasheng) with GG alleles and three materials (Kainong No.8, Xixiachangman and Weihaidunhuasheng) with AA alleles on the SNP A09_114690064 locus were used to perform expression analysis **(S2 Table in S2 File)**. We chose stage 7 [34] seeds of these materials based on the expression profile of peanut [35] which showed “Pattee 7 seed” (i.e. seed in the stage 7, [34]) with the highest expression of *ahFAD2A* in seeds of different developmental stages. At stage 7, the seeds are torpedo to round shaped, the seeds at the end of the embryonic axis are pink and the seeds at the other end are white to light pink [34]. Seeds that located at the other end of embryonic axis in stage 7 were carefully collected from the six materials for RNA extraction. About 0.1–0.2g frozen seeds were ground into powder rapidly and mixed with 1 ml TRIzol (Invitrogen) in a centrifuge tube. After homogenizing and incubating at room temperature for 5 min, 0.2 ml of chloroform were added to the mixture. The samples were shook by hand for 15 seconds, incubated at room temperature for 5 min, followed by centrifugation at 12000 rmp for 15 minutes at 4°C. The upper aqueous phase was carefully transferred to a clean centrifuge tube, then added the same volume of isopropanol and placed the samples at -20°C for 30 min. After centrifuging (12,000 rmp, 10 min, 4°C), the supernatant was removed, and the RNA pellet was washed with 1 ml 75% ethanol (prepared with DEPC water). The tubes were centrifuged at 7,500 rmp for 5 min at 4°C, then the top solution was discarded. The RNA pellet was vacuum-dried for 30 min and finally dissolved in 50 μL of DEPC water. A 1% (p/v) agarose gel was run to visualize the integrity of the RNA. The RNA was quantified using the absorbance at OD260/OD280 and OD260/OD230 nm measured with a NanoDrop 2000c spectrophotometer (Thermo Scientific, Waltham, MA, USA). Equal amounts (2 μg) of total RNA were reverse transcribed with Moloney murine leukemia virus reverse transcriptase (Fermentas). The quantitative reverse-transcriptase PCR (qRT-PCR) was performed with the Bio-Rad CFX96 Real-Time System (Bio-Rad, Hercules, CA, USA). Each reaction was performed in triplicate and in a 20-μL volume containing 10 μL 2× SYBR Green Mix, 2 μL forward primer (2μM), 2 μL reverse primer (2 μM), 4 μL cDNA working solution, and 2 μL RNase-free ddH_2_0. qRT-PCR conditions were performed using the following thermal program: 95°C for 10 min; followed by 40 cycles of 95°C for 15 s and 60°C for 50 s. The relative expression was calculated by using the 2^−ΔΔCt^ method and normalized by using the internal reference actin gene [36].The specific primers of qRT-PCR for *ahFAD2A* included *ahFAD2A*-qRTF: 5’-TGTTGTCTATGATCTCTTAGTGGC-3’, *ahFAD2A*–qRTR: 5’-GGGTATGGAAGCTTGTGGAAA-3’. Actin sequences were *AhActin*-F: 5’-TAAGAACAATGTTGCCATACAGA-3’, *AhActin*-R: 5’-GTTGCCTTGGATTATGAGC-3’.

## Results

### Phenotypic variations of fatty acids

The 250 lines of the Chinese peanut mini-core collection were planted at two locations in two years and the contents of eight fatty acids including C16:0, C18:0, C18:1, C18:2, C20:0, C20:1, C22:0 and C24:0 were measured from seeds harvested from the four environments. The continuous distributions of fatty acid phenotypic values for the mini-core accessions were shown in **S1 Fig in S1 File: Table 1**. The average coefficient of variation (CV) of four environments for each trait varied from 13.50 to 22.24% **(Table 1)**. The trait of C18:2 content showed the highest CV (22.24%), followed by C20:1 (21.81%) and C18:0 (21.03%). The trait of C16:0 content showed the lowest CV (13.50%). Similarly, the highest and lowest CV in single environment were observed for C18:2 content in E4 (30.15%) and C16:0 content in E1 (11.8%, **Table 1**). The broad-sense heritabilities (*H*^*2*^) for contents of C16:0, C18:0, C18:1, C18:2, C20:0, C20:1, C22:0 and C24:0 across environments were evaluated to be 73.88%, 80.50%, 93.98%, 93.57%, 83.50%, 90.65%, 80.70% and 77.55%, respectively (**Table 1**).

**Table 1:** Description of phenotypic analysis for the fatty acid traits in the association population under multi-environments.

Trait	Env.	Min (%)	Max (%)	Mean (%)	SD	CV (%)	*H*^*2*^ (%)
Palmitic acid	E1	8.19	15.81	11.39	1.34	11.80	73.88
(C16:0):	E2	7.87	14.27	10.89	1.40	12.86	
	E3	8.04	14.11	11.02	1.44	13.09	
	E4	7.16	13.81	10.44	1.69	16.23	
Stearic acid	E1	1.54	9.72	3.67	0.91	24.72	80.50
(C18:0)	E2	1.59	5.66	3.26	0.68	20.74	
	E3	1.77	5.07	3.41	0.61	17.93	
	E4	1.90	6.21	3.93	0.81	20.74	
Oleic acid	E1	34.47	64.85	47.81	6.53	13.67	93.98
(C18:1)	E2	35.09	63.98	48.40	7.34	15.17	
	E3	35.64	63.63	47.68	7.62	15.98	
	E4	38.39	71.73	53.16	8.86	16.67	
Linoleic acid	E1	17.63	41.69	31.35	5.45	17.39	93.57
(C18:2)	E2	16.75	42.97	31.10	6.24	20.06	
	E3	16.11	41.15	30.14	6.44	21.37	
	E4	11.04	38.55	25.26	7.62	30.15	
Arachidic acid	E1	1.01	2.79	1.50	0.23	15.44	83.50
(C20:0)	E2	0.93	2.34	1.53	0.22	14.31	
	E3	1.15	2.44	1.73	0.22	13.01	
	E4	1.05	2.51	1.78	0.26	14.67	
Gadoleic acid	E1	0.42	1.69	0.82	0.20	24.39	90.65
(C20:1)	E2	0.54	1.60	0.89	0.20	22.22	
	E3	0.74	1.86	1.09	0.23	20.86	
	E4	0.61	1.71	0.98	0.19	19.77	
Behenic acid;	E1	1.30	3.85	2.27	0.39	17.24	80.70
(C22:0)	E2	1.71	4.04	2.61	0.39	14.92	
	E3	1.69	4.27	3.16	0.41	13.06	
	E4	2.03	3.88	2.86	0.36	12.74	
Lignoceric acid	E1	0.58	2.19	1.18	0.25	20.77	77.55
(C24:0)	E2	0.73	2.18	1.30	0.25	18.90	
	E3	0.89	2.76	1.77	0.25	14.09	
	E4	0.97	2.30	1.59	0.23	14.73	

Env. represents environment. E1: Nanchong in 2015; E2: Wuhan in 2015; E3: Nanchong in 2016; E4: Wuhan in 2016. SD: standard deviation. CV: Coefficients of variation. *H*^2^ indicates the broad-sense heritability.

Correlation analysis showed that 15 trait pairs were pairwise significantly correlated with each other in four environments, 5 trait pairs were pairwise significantly correlated with each other in three environments, and 6 trait pairs were pairwise significantly correlated with each other in two environments (**Table 2**). Fourteen trait pairs were significantly negatively correlated, and 11 trait pairs were significantly positively correlated. The top five trait pairs are C18:1-C18:2 (range: -0.98 to -0.99; mean: -0.99), C16:0-C18:1 (range: -0.81 to -0.93; mean: -0.87), C16:0-C18:2 (range: 0.78 to 0.93; mean: 0.85), C18:0-C20:0 (range: 0.68 to 0.95; mean: 0.86), C18:0-C20:1 (range: -0.64 to -0.7; mean: -0.67).

**Table 2:** Correlation matrix among fatty acid components across four environments.

Trait	Env.	Palmitic acid (C16:0)	Stearic acid (C18:0)	Oleic acid (C18:1)	Linoleic acid (C18:2):	Arachidic acid (C20:0)	Gadoleic acid (C20:1)	Behenic acid (C22:0)
Stearic acid	E1	-0.07						
(C18:0)	E2	-0.15*						
	E3	-0.12*						
	E4	-0.38***						
Oleic acid	E1	-0.81***	-0.14*					
(C18:1)	E2	-0.84***	0.02					
	E3	-0.91***	0.08					
	E4	-0.93***	0.26***					
Linoleic acid	E1	0.78***	0.01	-0.98***				
(C18:2)	E2	0.82***	-0.11	-0.99***				
	E3	0.88***	-0.17**	-0.99***				
	E4	0.93***	-0.34***	-0.99***				
Arachidic acid	E1	-0.21**	0.86***	-0.06	-0.08			
(C20:0)	E2	-0.33***	0.68***	0.13*	-0.2**			
	E3	-0.08	0.95***	0.01	-0.11			
	E4	-0.4***	0.94***	0.24***	-0.33***			
Gadoleic acid	E1	-0.37***	-0.68***	0.38***	-0.33***	-0.44***		
(C20:1)	E2	-0.29***	-0.64***	0.24***	-0.19**	-0.54***		
	E3	-0.49***	-0.7***	0.47***	-0.42***	-0.63***		
	E4	-0.33***	-0.64***	0.37***	-0.32***	-0.52***		
Behenic acid	E1	0.05	0.09	-0.23***	0.12*	0.45***	0.3***	
(C22:0)	E2	-0.02	-0.06	-0.24***	0.2	0.31***	0.28***	
	E3	0.23***	0.09	-0.32***	0.24***	0.33***	0.05	
	E4	0.23***	0.06	-0.34***	0.27***	0.31***	0.07	
Lignoceric acid	E1	-0.37***	-0.28***	0.23***	-0.26***	0.06	0.69***	0.53***
(C24:0)	E2	-0.31***	-0.27***	0.08	-0.07	-0.04	0.57***	0.35***
	E3	-0.33***	-0.25***	0.18**	-0.19**	-0.13*	0.6***	0.23***
	E4	-0.37***	-0.15*	0.25***	-0.26***	0.07	0.62***	0.39***

*** indicate significance at *P* < 0.001

** indicate significance at *P* < 0.01

* indicate significance at *P* < 0.05.

### Detection of associated loci for fatty acids

A suitable association panel should encompass as much molecular diversity as can be reliably measured [37]. Genetic diversity of the Chinese peanut mini‐core collection was estimated using 105814 SNPs [27]. From the average level of the whole mini-core collection, the major allele frequency, gene diversity and PIC were 0.75, 0.32, and 0.26, respectively.

**Fig 1:**
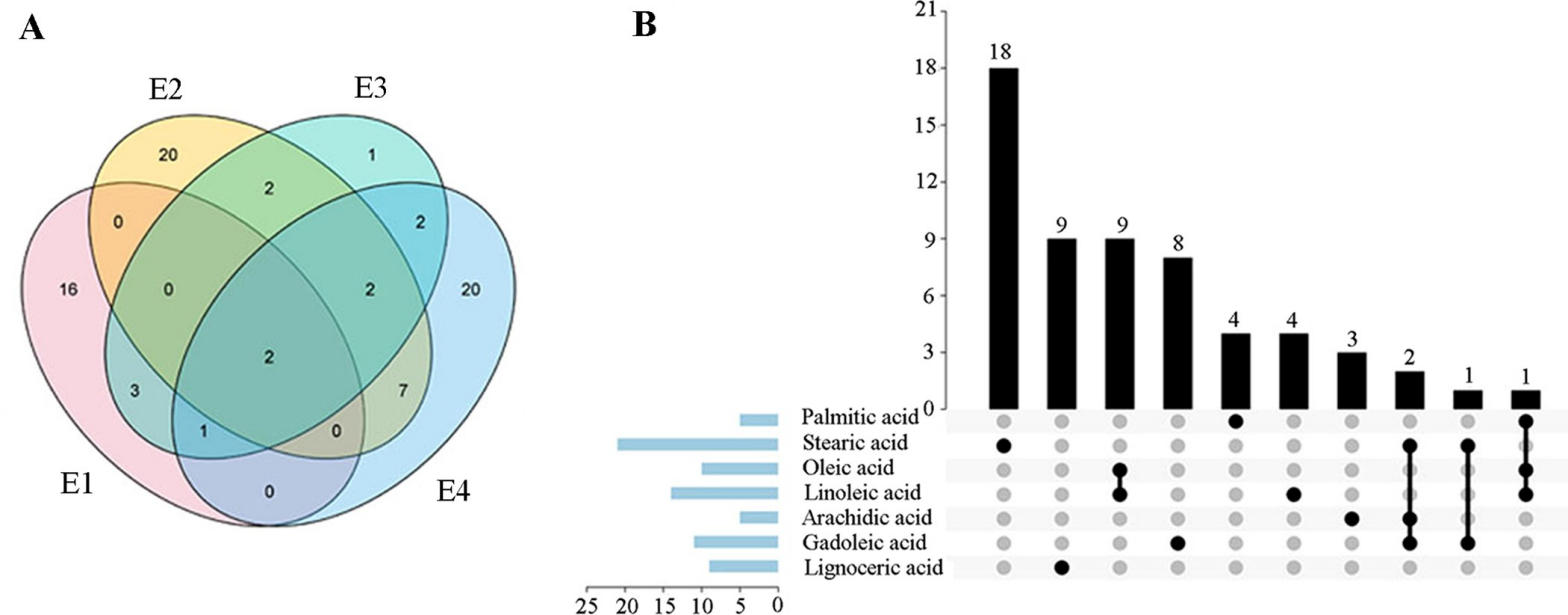
Overview of the significant associations identified for the fatty acid components (*P* < 4.73×10^−7^). (A) Distribution and overlap of identified associated loci for fatty acid traits under “E1-E4” environments. (B) Profile of associations for individual traits or co-localized traits. The vertical column diagram shows the number of the associated loci for individual or multiple traits; the interactive plot shows that these traits identified same associated loci; the horizontal column diagram shows the number of associated loci for each trait. E1 refers to Nanchong in 2015; E2 refers to Wuhan in 2015; E3 refers to Nanchong in 2016; E4 refers to Wuhan in 2016.

**Fig 2:**
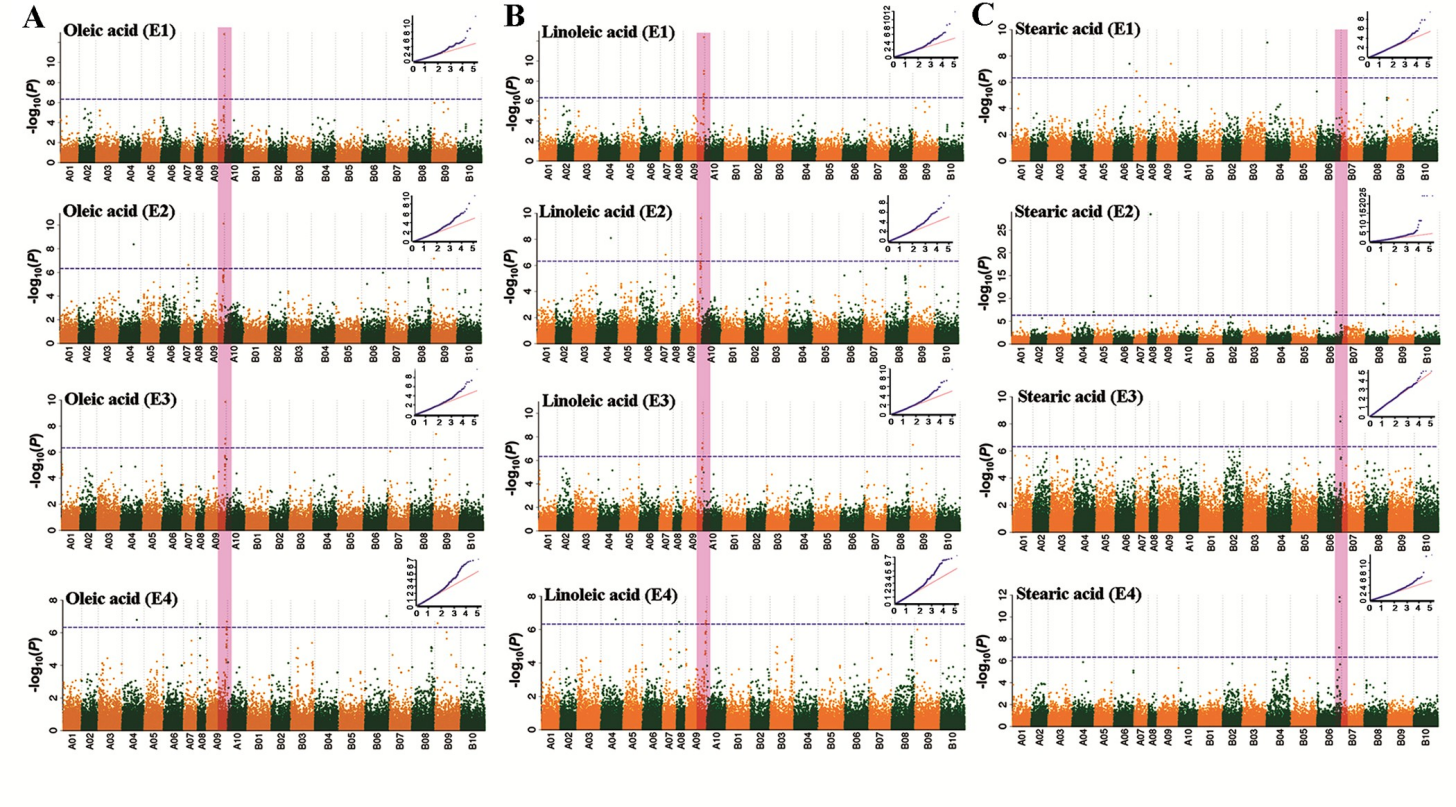
GWAS association loci for oleic acid, linoleic acid and stearic acid in the Chinese peanut mini-core collection under four environments. (A) Manhattan plots and quantile-quantile plots for oleic acid (C18:1). Negative log_10_(*P*) values from a genome-wide scan are plotted against position on each of twenty chromosomes. The horizontal dashed lines indicate the genome-wide significance threshold (-log_10_4.73×10^−7^ = 6.33). (B) Manhattan plots and quantile-quantile plots for linoleic acid (C18:2). (C) Manhattan plots and quantile-quantile plots for stearic acid (C18:0). The horizontal coordinates of quantile-quantile plots represented expected -log_10_(*P*), and the vertical coordinates of quantile-quantile plots represented observed -log_10_(*P*).

Association analysis showed that fatty acids had been identified 22 associated loci in E1, 33 associated loci in E2, 12 associated loci in E3 and 34 associated loci in E4 (*P* < 4.73×10^−7^, **Fig 1A, S3 Table in S2 File**). The larger number of associated loci for single trait in each environment was 14 for C18:0 content in E2, 8 for C18:2 content in E4 and 8 for C20:1 content in E2 and E4. However, no significant associated SNP for C22:0 content was identified in all environments (**S3 Table in S2 File**). For other single fatty acid components in four environments, a total of 5, 21, 10, 14, 5, 11 and 9 non-redundant associated SNPs were detected for C16:0, C18:0, C18:1, C18:2, C20:0, C20:1 and C24:0, respectively (**Figs 1B and 2, S3 Table in S2 File, S2-S6 Figs in S1 File**).

As mentioned above, a total of 75 significantly associated SNPs for fatty acids were detected in Chinese peanut mini-core collection (**Fig 1A, S3 Table in S2 File**). Of which, 19 loci were detected in at least two environments, including one loci for C18:0, six loci each for C18:1, C18:2, C20:1(**Fig 1A, S4 Table in S2 File**). The associated marker A09- 115528661 for C18:1 and C18:2 was consistently identified across four environments. The associated marker A09-114106219 for C18:1 content was repeatedly detected in “E3, E4”, and for C18:2 content in “E2, E3 and E4”. Similarly, the SNP B09- 15848169 was associated with C18:1 content in three environments, and C18:2 content in two environments.

A panel of thirteen SNP loci were detected to be co-associated with two or three fatty acid traits (**Fig 1B, S5 Table in S2 File**). Among them, ten markers were significantly associated with both C18:1 and C18:2 contents and seven of them were associated with both traits in multiple environments. Marker A08-13807926 was significantly associated with C16:0, C18:1, C18:2. Markers B06-125245720 and B06-125302832 were significantly associated with C18:0, C20:0 and C20:1, and marker B06-122677790 was significantly associated with C18:0, C20:1. The common associated markers for multi-traits identified by association analysis were consistent with the high phenotypic correlations among these traits.

A set of eight associated markers were detected high phenotypic variance explained (PVE, >10%), which were distributed on the A09, B04 and B06 chromosomes **(S6 Table in S2 File)**. Among which, markers A09-114106219, A09-115528661 for C18:1 and C18:2, and B06-122677790 for C18:0 showed major PVE in multiple environments (**Table 3**), while the other five loci have major PVE for fatty acid in specific environments.

**Table 3:** The major associated loci identified in multiple environments.

SNP Position	Reference	Tother	Trait	PVE	Env.	P value
A09-114106219	G	A	Oleic acid (C18:1)	40.48%	E3	6.64
				39.05%	E4	6.35
			Linoleic acid (18:2)	36.20%	E2	6.87
				38.22%	E3	7.02
A09-115528661	C	T	Oleic acid (C18:1)	8.20%	E3	9.86
				12.60%	E4	6.69
			Linoleic acid (C18:2)	12.06%	E2	9.63
				8.40%	E3	10.02
				10.50%	E4	7.08
B06-122677790	G	A	Stearic acid (C18:0)	18.72%	E3	7.23
				23.01%	E4	7.20

Env. represents environment. E2: Wuhan in 2015; E3: Nanchong in 2016; E4: Wuhan in 2016.

### Candidate genes underlying stable major associated loci

Among the associated loci with high phenotypic variance explained, three SNPs (A09-114106219, A09-115528661, B06-122677790) were stable (**Fig 2, Table 3**). The marker A09-114106219 was identified with ~40% PVE for C18:1 content in two environments, similar for C18:2. The marker A09-115528661 averagely explained more than 10% phenotypic variation for C18:1 content in two environments and for C18:2 content in three environments. The marker B06-122677790 for C18:0 content was identified with ~20% PVE in E3 and E4. The results suggested that the genomic regions on A09 and B06 might contain important loci/genes regulating the fatty acids in peanut.

Total 21 candidate genes involved in fatty acid biosynthesis pathway were identified in the genomic regions underlying the three stable major associated loci **(Table 4)**. The *Aradu*.*G1YNF* gene is predicted to encode fatty acid desaturase 2 (*FAD2A*) which is involved in the triacylglycerol biosynthesis pathway and has previously been identified as the key gene for oleic and linoleic acid [38, 39]. Seven genes were predicted to encode proteins involved in fatty acid elongation pathway, three genes were predicted to encode proteins involved in fatty acid synthesis pathway, and three genes were predicted to encode proteins in triacylglycerol and fatty acid degradation pathway. Five genes were predicted to encode proteins in phospholipid synthesis pathway, and a single gene each encoded proteins which involved in suberin synthesis and sphingolipid biosynthesis pathways.

**Table 4:** Candidate genes underlying stable major associated loci on chromosome A09 and B06.

Trait	Associated SNP	Position in diploid genome		Peanut gene ID	Position of genes in tetraploid genome (AABB)			Functional annotation
		Chr.	Candidate genomic region		Chr.	Start	End	
Oleic acid,	A09-114106219	A09	112806219–114228661	Aradu.V1XA0	Arahy.09	112552418	112559357	ABC transporter
Linoleic acid				Aradu.B3QR0	Arahy.09	112812745	112815516	Acyl-ACP thioesterase
(C18:1, C18:2)				Aradu.42SQT	Arahy.09	112810188	112822568	Phosphatidylserine synthase
				Aradu.6NR6S	Arahy.09	113004585	113009189	lipid Phosphatidate phosphatase
				Aradu.M3B1E	Arahy.09	113010087	113014838	lipid Phosphatidate phosphatase
	A09-114106219/A09-115528661	A09	114228661–115406219	Aradu.78G42	Arahy.09	113810264	113811996	Glycolipid transfer protein
				Aradu.RSF6Z	Arahy.09	113818849	113822654	Dihydrolipoamide acetyltransferase
				Aradu.G1YNF	Arahy.09	114172195	114175899	Fatty acid desaturase 2
				Aradu.35TX9	Arahy.09	114301150	114304504	Lipoate synthase
	A09-115528661	A09	115406219–116828661	Aradu.MK5FJ	Arahy.09	115951768	115954748	Phosphatidylglycerol-phosphate transferase
				Aradu.C03NZ	Arahy.19	151700249	151700563	Lipid transfer protein
				Aradu.WM0EZ	Arahy.09	116399973	116400287	Lipid transfer protein
				Aradu.P2T2I	Arahy.19	151713901	151714215	Lipid transfer protein
				Aradu.57IQ7	Arahy.09	116427681	116427995	Lipid transfer protein
				Aradu.37CBG	Arahy.19	151709284	151709726	Lipid transfer protein
				Aradu.PYT5N	Arahy.19	151763530	151763844	Lipid transfer protein
				Aradu.3DX6E	Arahy.09	116508816	116509847	Dienoyl coA isomerase
Stearic acid (C18:0)	B06-122677790	B06	121377790–123977790	Araip.N3W37	Arahy.16	136366061	136371271	NAD-dependent glycerol-3-phosphate dehydrogenase

### Allelic effect for C18:1 and C18:2 contents

**Fig 3:**
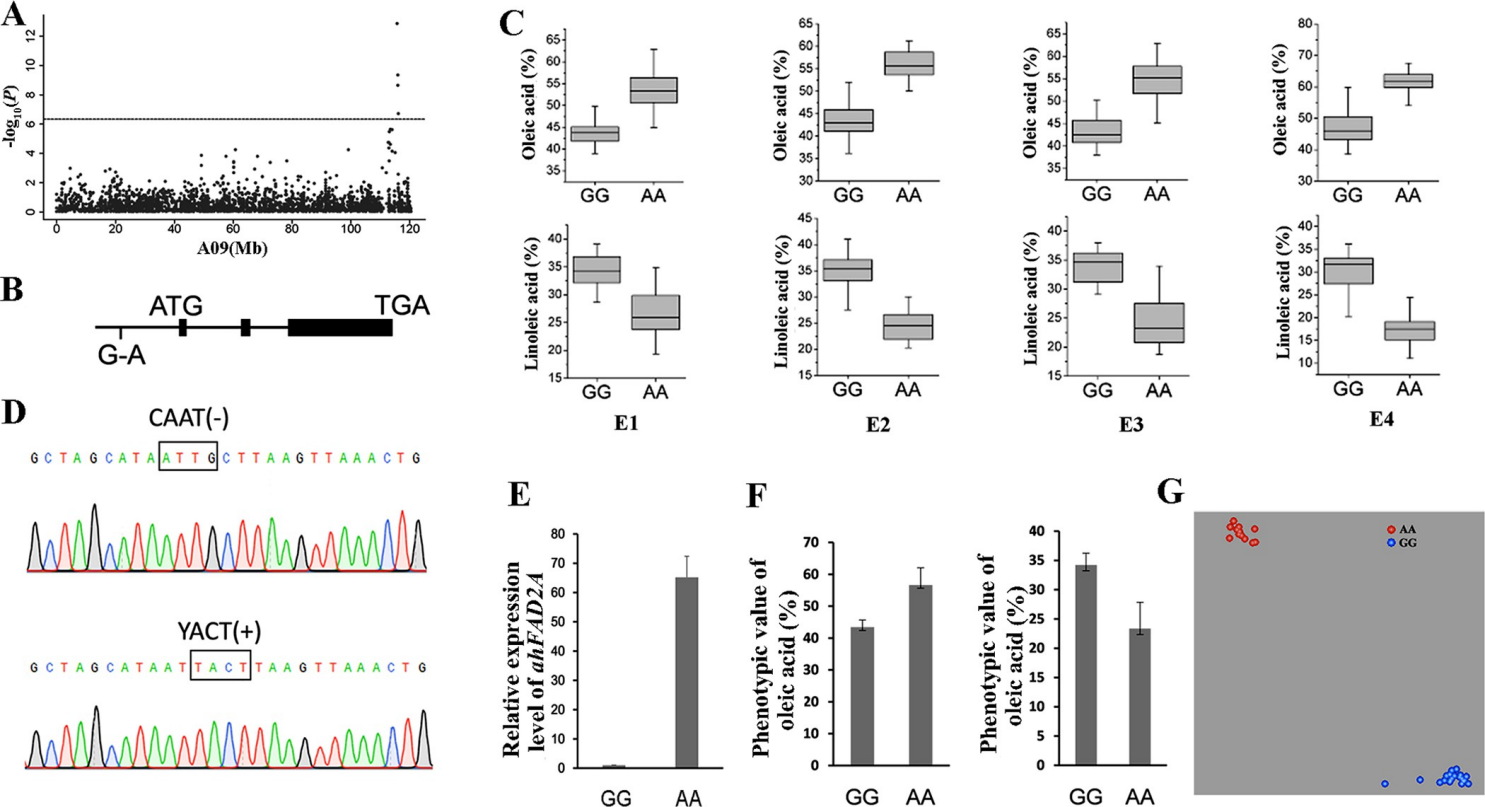
Genetic analysis of oleic acid and linoleic acid on A09 and validation of the diagnostic marker. (A) Take the Manhattan plots of A09 chromosome for oleic acid (C18:1) in E1 environment as an example. (B) The diagram of SNP (G-to-A) in the promoter region of *ahFAD2A*. (C) The allelic effect at A09-114690064 for oleic acid (C18:1) and linoleic acid (C18:2) under four environments. For each trait, the boxes with GG alleles and AA alleles were significant different according to Tukey’s Multiple Comparison Test (*P* < 0.05) (D) The partial sequence diagrams included the diagnostic locus of the SNP in the promoter of *ahFAD2A* after amplifying by PCR and sequencing. The diagnostic SNP locus changed the cis-element of CAAT (-) to enhancer-like module YACT (+). (E) qRT–PCR result of *ahFAD2A* in seeds at stage 7. (F) Phenotypic differences between accessions carrying different alleles of the SNP A09-114690064 of two extreme trait groups of peanut. *P*<0.001, Student’s t-test. (G) Scatter plots using KASP marker genotyping.

The correlation between base variation in the candidate genes and phenotypic variation were further analyzed in the peanut mini-core population. Unfortunately, we did not find the existing non-synonymous SNP in the candidate genes, but we found eight SNPs in the upstream of the candidate genes underlying the three stable major associations of A09-114106219, A09-115528661 and B06-122677790. The allelic effects of these eight SNPs on target fatty acid components were investigated under “E1-E4” environments. We found one SNP A09-114690064 in the promoter region of *Aradu*.*G1YNF* (*ahFAD2A*) which showed that the AA genotype had significant higher (*P* < 0.05) oleic acid content than that with the GG genotype, while the linoleic acid content showed opposite values in the association panel under four environments (**Fig 3A–3C**).

PCR products sequencing further confirmed that the SNP which leads to a G-A base change at A09-114690064 in the promoter region of *ahFAD2A* did exist (**Fig 3D, S7 Fig in S1 File**). Functional element analysis of promoter found that the base variation changes the cis-element CAAT (-) to YACT (+) (**Fig 3D**). The previously study indicated that tetranucleotide CACT element in the distal region of C4 plants promoter acts as an enhancer-like expression module and sufficient for expression [40], while the CAAT elements is insufficient for expression (to initiate transcription) [41]. To examine the effect of the base change in the cis-element of promoter region on gene expression, quantitative reverse-transcriptase PCR (qRT–PCR) were performed in seeds at stage 7 [34]. The results showed that *ahFAD2A* displayed dozens of times higher expression level in the AA genotype than in the GG genotype (**Fig 3E, S2 Table in S2 File**), suggesting this SNP played an important role in transcriptional regulation.

The SNP A09-114690064 was targeted for cost-effective KASP marker development and further validate the effect. The KASP marker was used to genotype 22 accessions with low oleic and high linoleic acid content and 13 accessions with high oleic and low linoleic acid content (**Fig 3F and 3G**). The results showed that the diagnostic marker, Aradu-A09-114690064, amplified the AA alleles in the high oleic and low linoleic acid accessions while the GG alleles in the low oleic and high linoleic acid accessions (**Fig 3F and 3G; S7 Table in S2 File**). Based on the results above, the Aradu-A09-114690064 could be used as an effective diagnostic marker for marker-assisted selecting breeding of high oleic peanut varieties.

## Discussion

The Chinese peanut mini-core collection was selected from 6,390 Chinese peanut collection based on their basic data (botanical type, geographical origin, etc.) and 15 character data (morphological, agronomic and quality traits, etc.) and through cluster analysis [42, 43]. Previous studies evaluated the genetic diversity of the mini‐core collection using SSR markers [44, 45]. In this study, 105814 SNPs were used to estimate the genetic diversity of this population. An average major allele frequency of 0.75, the mean genetic diversity of 0.32, and the mean PIC of 0.26 were observed in the mini‐core collection containing 250 germplasm resources. Using this population, genome-wide association analysis was performed to elucidate genetic basis of fatty acids. The identified significant associations included 5 for C16:0 content, 21 for C18:0 content, 10 for C18:1 content, 14 for C18:2 content, 5 for C20:0 content, 11 for C20:1 content, 9 for C24:0 content. Correlation analysis showed a high negative correlation between C18:1 and C18:2 content (mean correlation coefficient: -0.99). Furthermore, among the identified associated loci with apparent pleiotropic effects, 10 were found for both C18:1 and C18:2 contents at different positions in the genome. The correlation coefficients among C18:0, C20:0 and C20:1 were high (C18:0-C20:0, mean: 0.86; C18:0-C20:1, mean: -0.67, and C20:0-C20:1, mean: -0.53). C18:0 and C20:1 content were detected three identical association loci, two of which were also associated with C20:0. The results showed that the traits with high correlation coefficients in fatty acids tended to have the same associated loci. The reason may be that these fatty acids have upstream-downstream relationship in the metabolic pathway, thus showing as a single locus (gene) that affects the composition of different fatty acids simultaneously. For example, oleic acid is the direct substrate for linoleic acid synthesis, and the *FAD2A* gene mutation leads to both increased oleic acid and decreased linoleic acid.

Some of the associated loci identified in this study were consistent with QTLs obtained from segregating populations. The major loci associated with oleic acid and linoleic acid on A09 identified in this study were located in or near the QTL regions identified in three segregation populations. These linkage analysis QTLs were *S_mqOA_a09* and *S_mqLA_a09* in S population (SunOleic 97R× NC94022) [4]; *T_mqOA_a09* and *T_mqLA_a09* in T population (Trifrunner×GT-C20) [4], *qOle-A09-1* and *qLin-A09-1* in FA population (ICGV 06420×SunOleic 95R) [16]. In addition, the repeatedly detected associated site of B09-15848169 for oleic acid and linoleic acid in this study was also consistent with the QTLs identified in segregating populations. These QTLs included *S_mqOA_b09-1* and *S_mqLA_b09-1* in S population (SunOleic 97R×NC94022) and QTLs *qOle-B09* and *qLinB09* in FA population (ICGV06420×SunOleic 95R) [4, 16]. The closest flanking markers of peak QTLs of the two populations were SSR markers developed by *ahFAD2B*. Moreover, six fatty acid-related genes were found underlying the associated SNP B09-15848169, including *FAD2B* encoded by *Araip*.*WI5IC*. Moreover, 19 associated loci for fatty acids detected by GWAS were repeatedly identified in at least two environments. The associated loci were identified in different genetic backgrounds or multiple environments, confirming that these loci were stable and reliable.

In previous studies, a SNP (G/A) in *ahFAD2A* was identified at the 448^th^ nucleotide of the coding region and the allele mutational peanut showed higher oleic acid content. Previous studies have shown that the 448G>A mutation does not cause significant difference in transcription level but has significant effect on the protein level between high (448A) and normal (448G) oleic acid peanut seeds [38, 46], showing that ah*FAD2A* has a cellular regulatory mechanism by regulating protein level in peanut. In this study, we identified a new variation (G/A) in the promoter region of *FAD2A* in peanut, which changes cis-element CAAT (-) to enhancer-like expression module YACT (+). Shirsat et al (1989) [41] showed that the CAAT elements in the promoter was insufficient to initiate transcription for expression, but the CACT element in the promoter acted as an enhancer-like expression module and was sufficient for expression in plants. Our qRT–PCR results showed that the expression level of *ahFAD2A* displayed dozens of times higher in the accessions with YACT (+) module than that in the accessions with CAAT (-) element in promoter. This variation of G-A in the promoter led to a remarkable change in the transcription level, suggesting that there was also a transcriptional regulatory mechanism regulating *FAD2A* expression in peanut. The function of CAAT (-) / YACT (+) element in the promoter of *ahFAD2A* needs to be ultimately confirmed by follow-up expression analysis and transcription factor-promoter interaction analysis.

Using sequencing-based trait mapping and effect estimation of nucleotide polymorphisms, researchers have identified some loci/genes associated with target traits as well as sequence variations for genes correlated with phenotypic variation [19]. This approach played an important role in the study of genetic regulation mechanism, candidate gene screening and the development of diagnostic markers for target traits. Using this method, diagnostic markers for rust resistance, late leaf spot resistance, bacterial wilt, seed (pod) size and weight have been developed in Peanut [27, 47, 48]. In this study, the allele diversity of the SNP A09_114690064 exhibited stable correlation with the phenotype variation in the associated population across environments. In addition, the marker Aradu_A09_114690064 amplified the AA alleles in the high oleic and low linoleic acid accessions (oleic acid content range: 54.68%-64.61%, linoleic acid content range: 17.65%-25.64%), and the GG alleles in the low oleic and high linoleic acid accessions (oleic acid content range: 39%-47.4%, linoleic acid content range: 30.01%-37.16%) (**Fig 3F, S7 Table in S2 File**). These results showed the validity and stability of the allelic effect. In general, KASP markers have many advantages, such as high accuracy, good flexibility and low cost. The KASP diagnostic marker based on A09_114690064 locus in this study could be useful to facilitate high-oleic peanut breeding.

## Supporting information

S1 File (PDF)

S2 File (XLSX)
