# Supplementary material for: Genetic dissection of fatty acid components in the Chinese peanut (Arachis hypogaea L.) mini-core collection under multi-environments
Source: PLoS One. 2022 Dec 30;17(12):e0279650. doi: 10.1371/journal.pone.0279650 (PMC9803190; doi:10.1371/journal.pone.0279650)
Supplement: S1 File — (PDF) [file pone.0279650.s001.pdf]

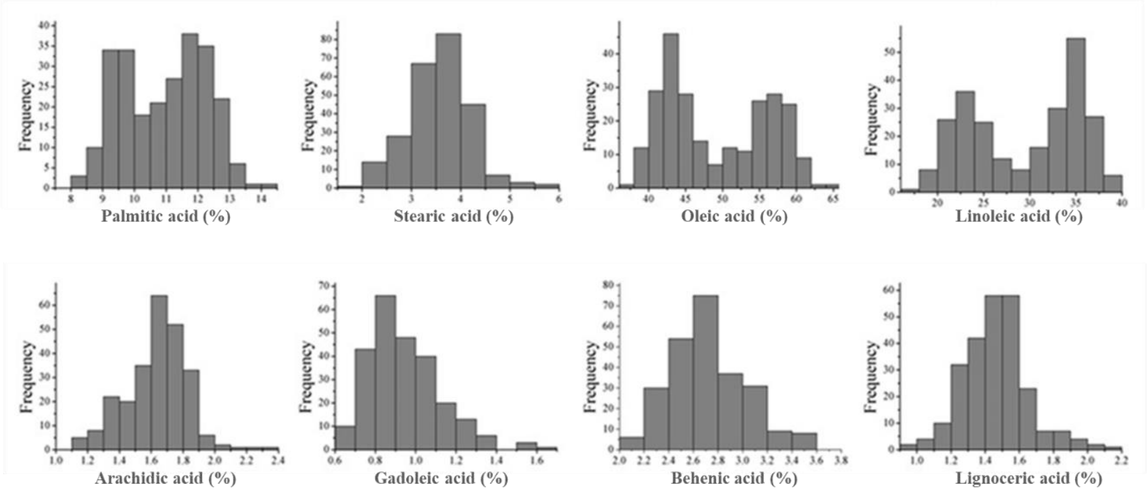

S1 Fig. Phenotypic distribution of the eight fatty acid traits in the Chinese peanut mini-core collection. *x*-axis represented average percentage range of four environments of fatty acids and y-axis showed the number of individuals in the peanut panel.

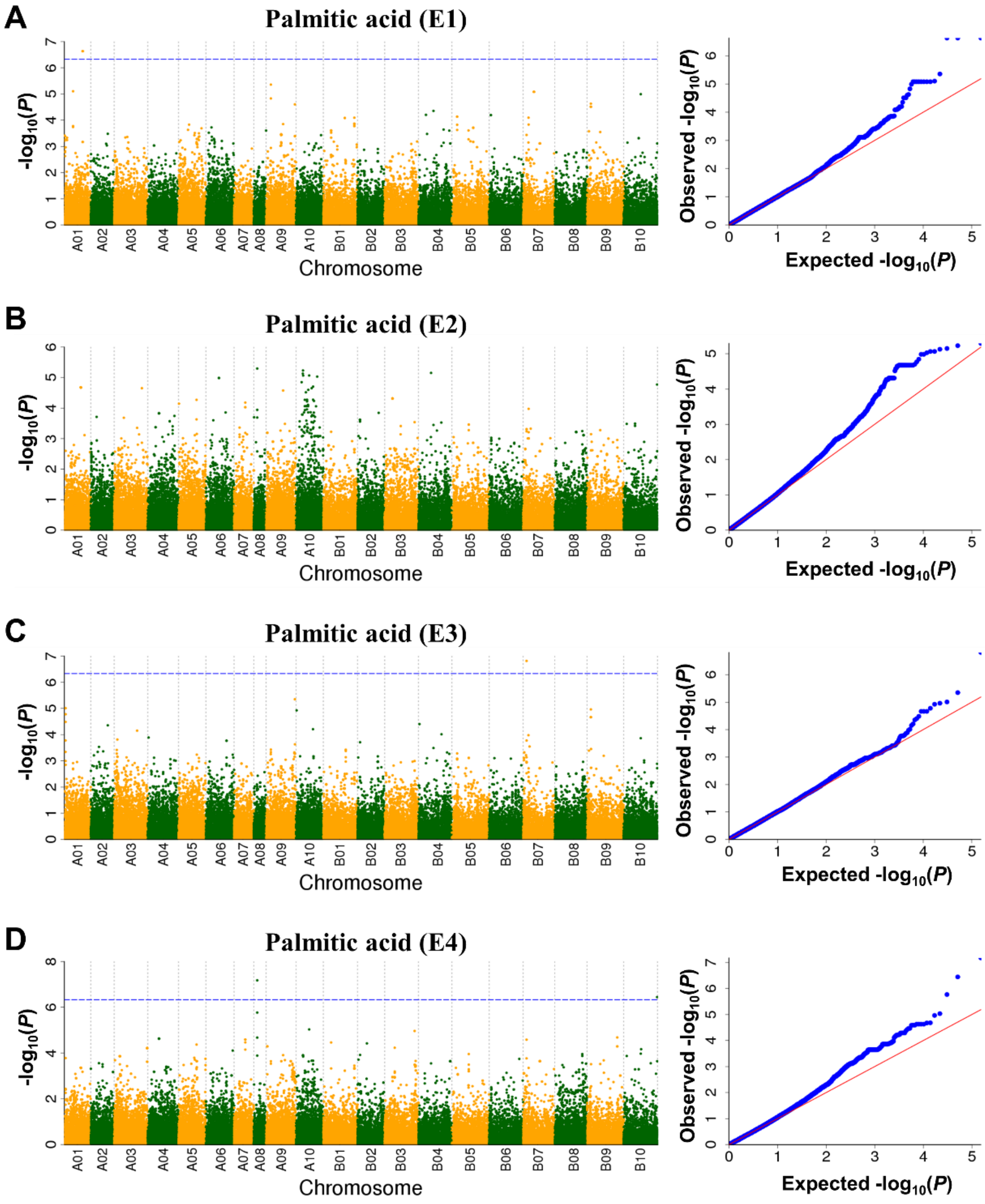

S2 Fig. Manhattan plots and quantile-quantile plots of GWAS for palmitic acid (C16:0) under four environments.

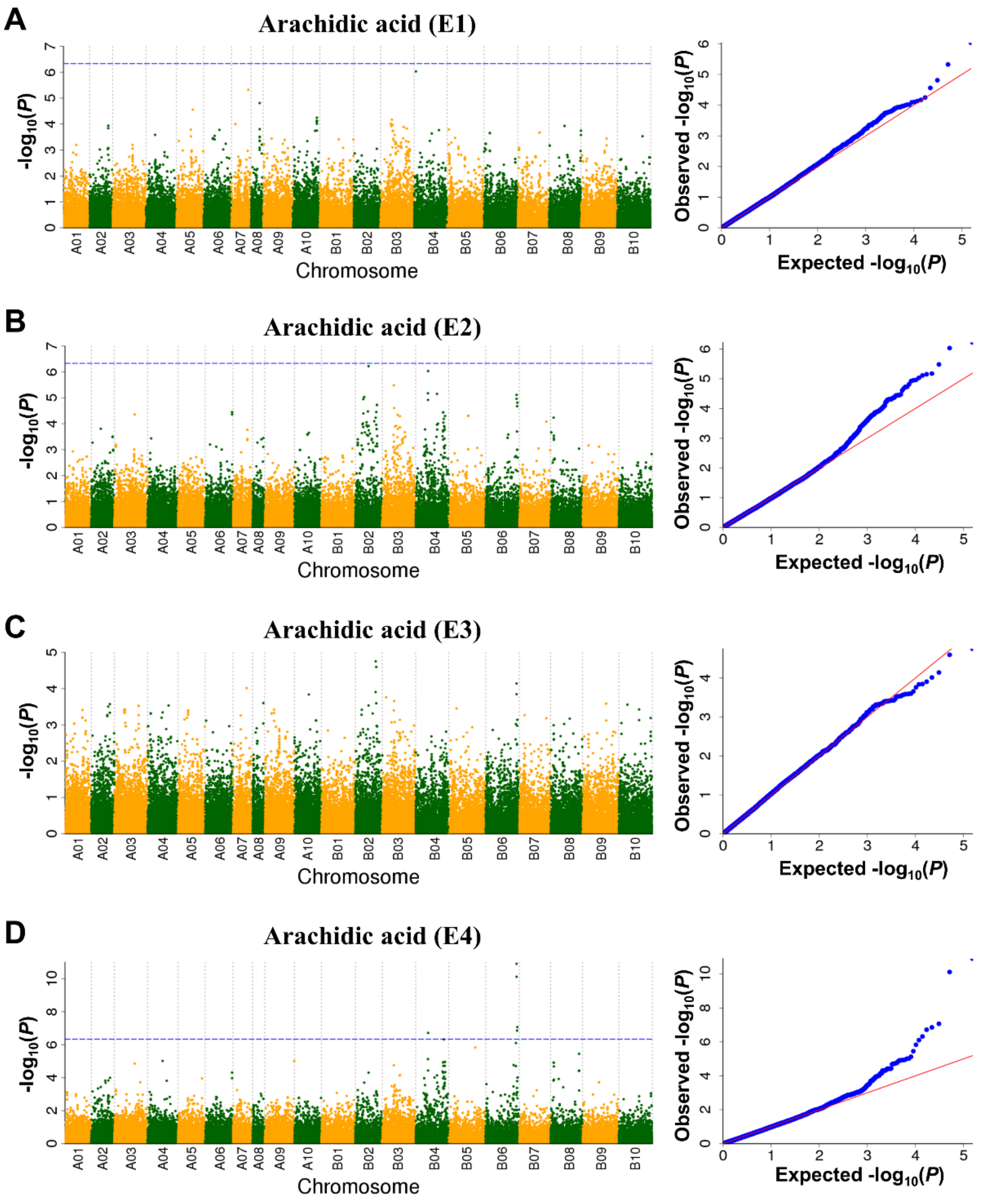

S3 Fig. Manhattan plots and quantile-quantile plots of GWAS for arachidic acid (C20:0) under four environments.

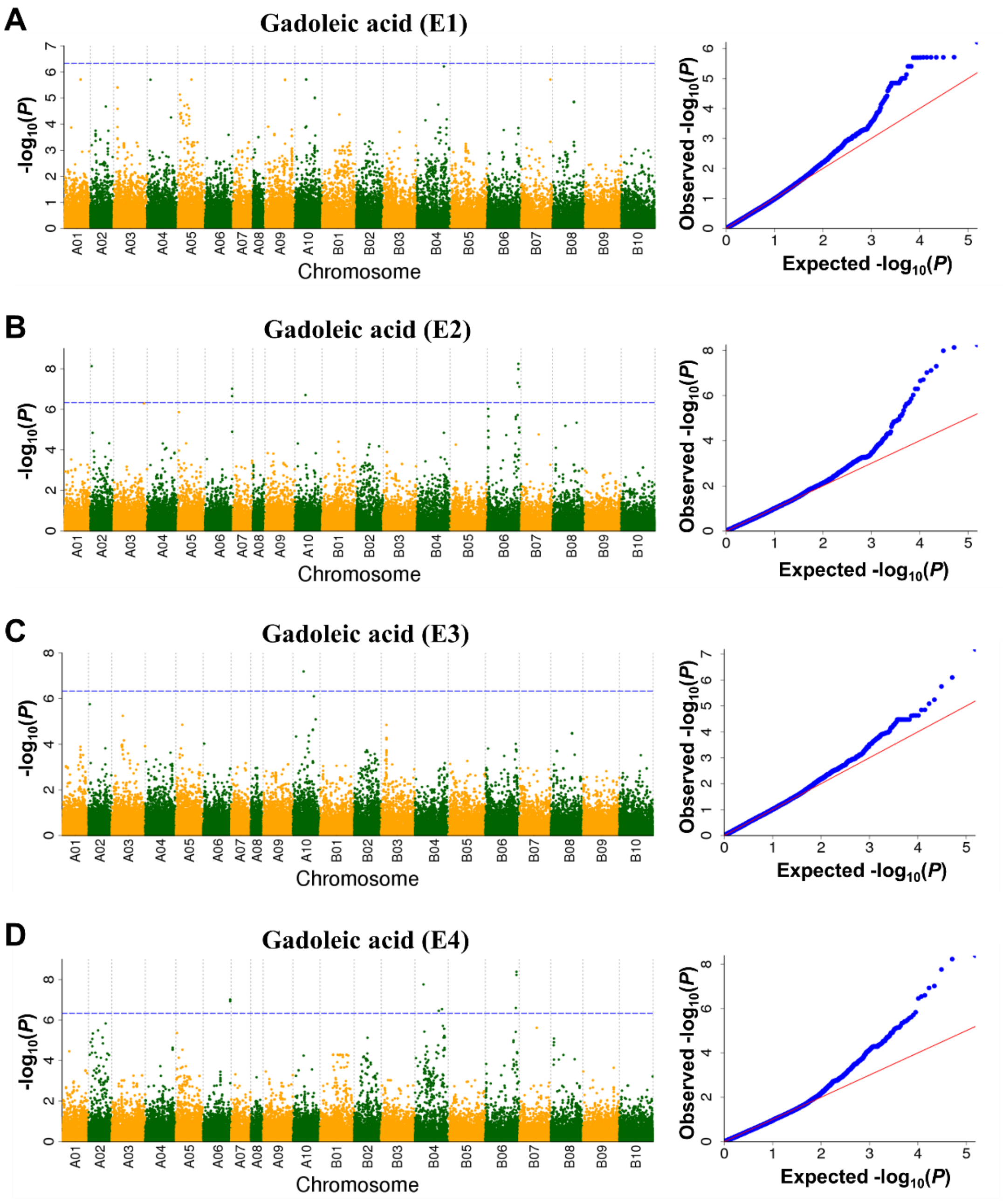

S4 Fig. Manhattan plots and quantile-quantile plots of GWAS for gadoleic acid (C20:1) under four environments.

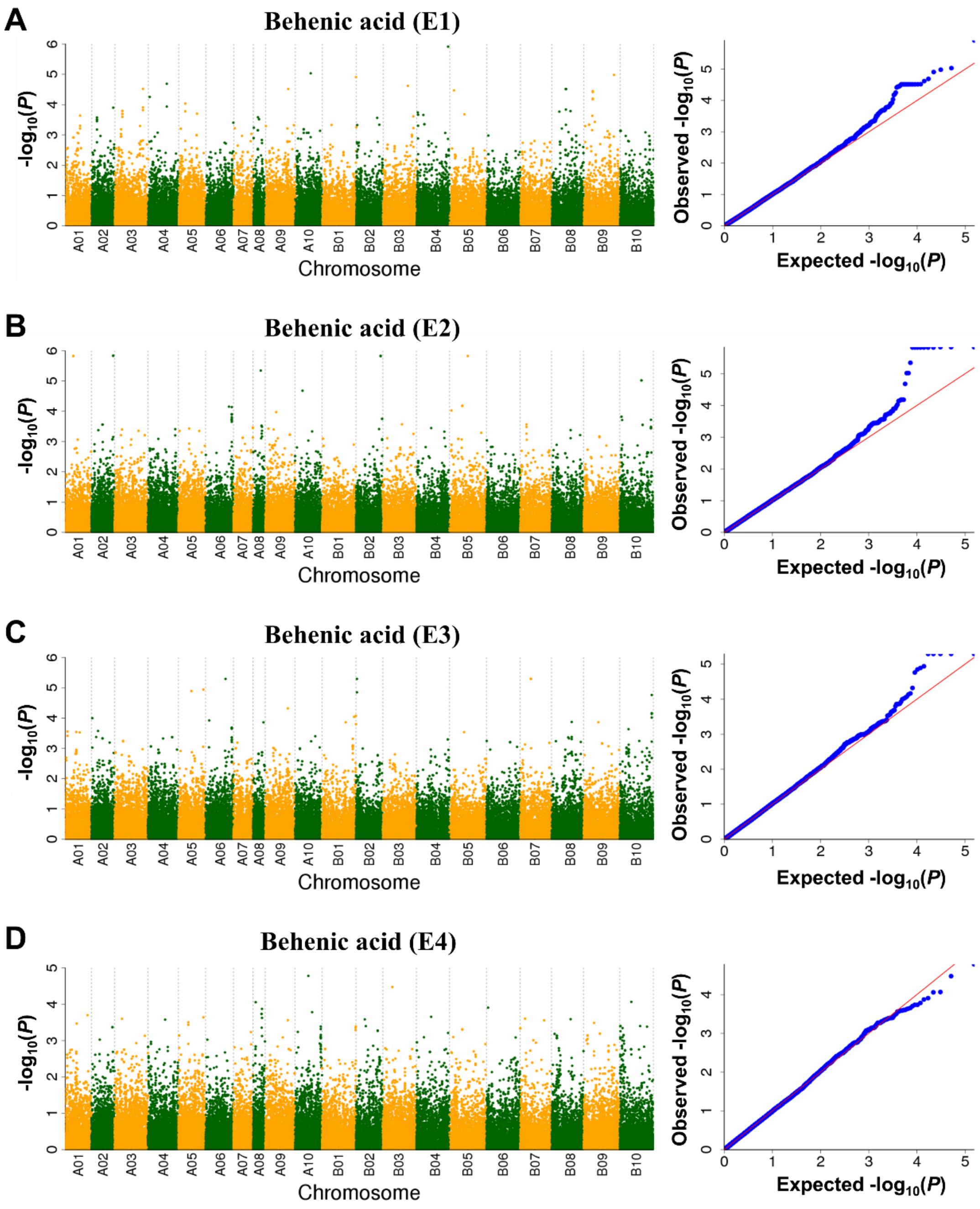

S5 Fig. Manhattan plots and quantile-quantile plots of GWAS for behenic acid (C22:0) content under four environments.

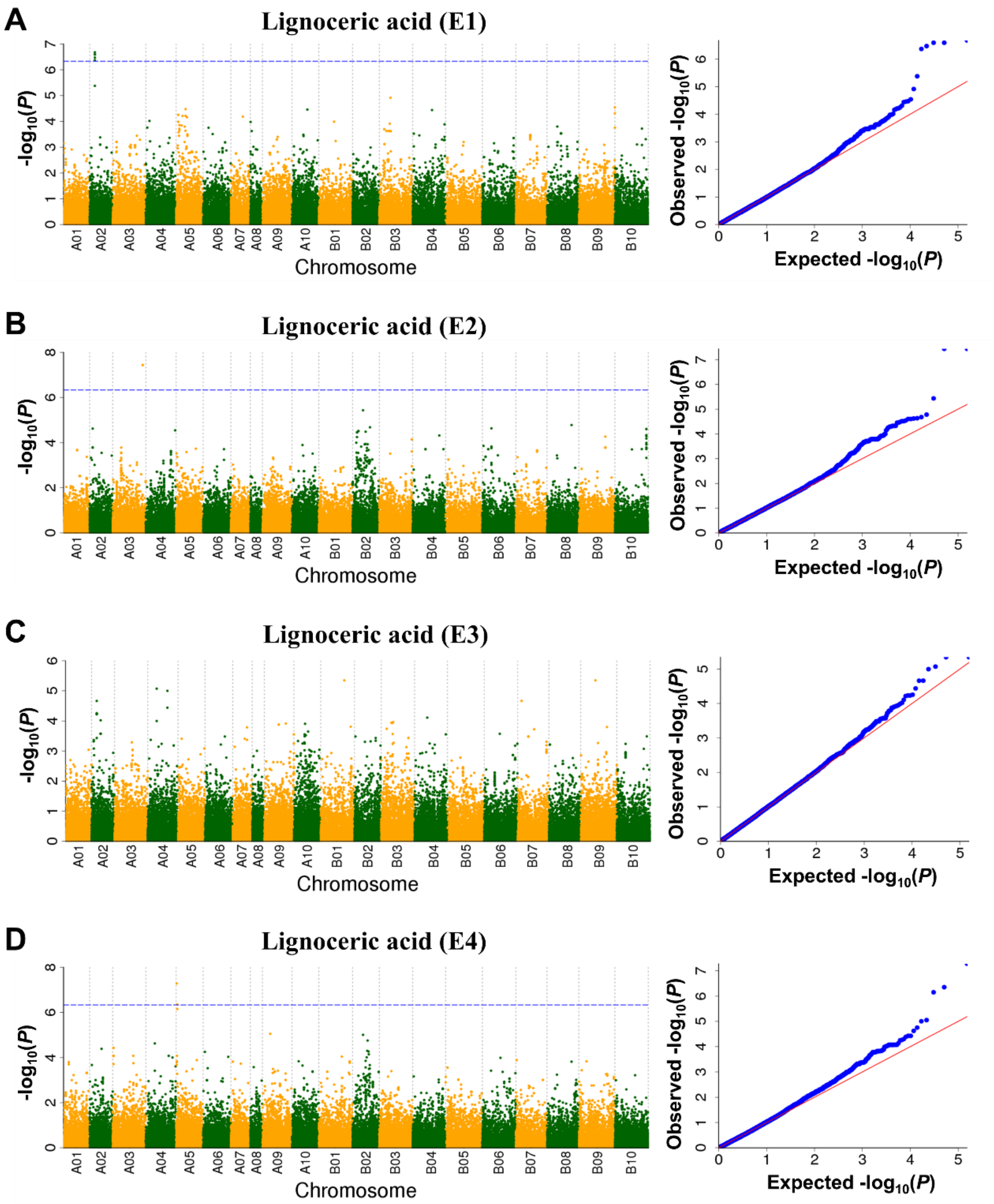

S6 Fig. Manhattan plots and quantile-quantile plots of GWAS for lignoceric acid (C24:0) under four environments.

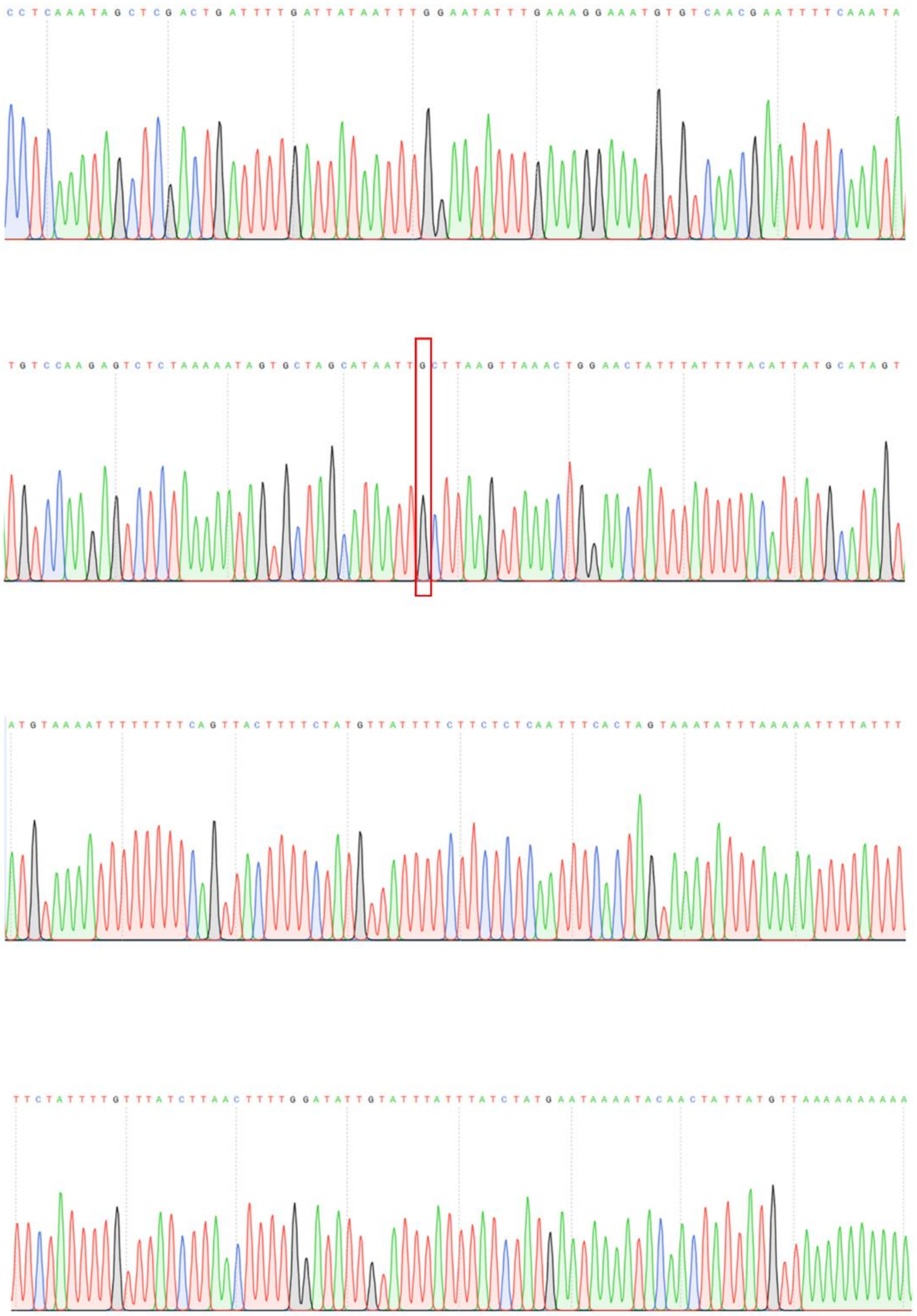

S7 Fig. A sequencing fragment including the A09-114690064 locus. The red box showed the position of this nucleobase.
